# First record of the male, new distribution and re-description of *Indopadillayordon* (Araneae, Salticidae)

**DOI:** 10.3897/BDJ.13.e159695

**Published:** 2025-08-18

**Authors:** Duc-Toan Vu, Dinh-Sac Pham

**Affiliations:** 1 Faculty of Agriculture and Forestry, Tay Bac University, Quyet Tam ward, Son La, Vietnam Faculty of Agriculture and Forestry, Tay Bac University, Quyet Tam ward Son La Vietnam; 2 Vietnam National Museum of Nature (VNMN), Vietnam Academy of Science and Technology (VAST), 18 Hoang Quoc Viet, Cau Giay, Hanoi, Vietnam Vietnam National Museum of Nature (VNMN), Vietnam Academy of Science and Technology (VAST), 18 Hoang Quoc Viet, Cau Giay Hanoi Vietnam

**Keywords:** new description, morphology, Salticidae, Vietnam

## Abstract

**Background:**

A total of seven known *Indopadilla* species are known from Vietnam: *I.abramovi* Logunov, 2024; *I.annamita* (Simon, 1903); *I.cuc* Wang, Li & Pham, 2023; *I.phantoani* Hoang & Zhang, 2023; *I.retsivn* Hoang, Phan & Vo, 2024; *I.yokdon* Hoang, Phan & Vo, 2024; and *I.namkarensis* Hoang, 2025. Amongst these, *I.yokdon* was previously known only from the female holotype collected in Yok Don National Park, Buon Don District, Dak Lak Province, Vietnam. In contrast, the remaining species were described, based on both male and female specimens. In the present study, the male of *I.yokdon* is described for the first time and the females are re-described, based on additional material collected from Dak Nong and Son La Provinces, Vietnam.

**New information:**

Our surveys yielded records of three individuals of *Indopadillayokdon* from two localities: one male and one female from Son La Province and one female from Dak Nong Province. This paper provides a first detailed description of the male and a re-description of the female. A morphological comparison between the sexes is also presented to support future taxonomic and phylogenetic studies. Additionally, the record from Son La Province extends the known distribution range of the species.

## Introduction

The jumping spider genus *Indopadilla* Caleb & Sankaran, 2019, currently comprises 22 valid species mainly distributed in South-East Asia (WSC 2025). To date, seven *Indopadilla* species have been recorded from Vietnam: *I.abramovi*, *I.annamita*, *I.cuc*, *I.namkarensis*, *I.phantoani*, *I.retsivn* and *I.yokdon* (Simon 1903, Żabka 1988, Caleb et al. 2019, Hoang et al. 2023, Wang et al. 2023, Hoang et al. 2024, Hoang 2025). Of these, six species are known from both sexes, while *I.yordon* was previously known only from a single female specimen (WSC 2025).

*Indopadillayokdon* was first described, based on a single female specimen from Yok Don National Park, Dak Lak Province, Vietnam (Hoang et al. 2024). The examination of specimens collected from rocky mountain tropical forests revealed one male and one female of *Indopadilla* co-occurring at a site in Son La Province, as well as one additional female from Dak Nong Province. Based on somatic and genital features, all three specimens were identified as *I.yokdon*. The male specimen exhibits carapace, cheliceral and legs morphology similar to that of the females and its palpal structures are consistent with the diagnostic features of the genus *Indopadilla*. Accordingly, the male and female specimens were matched and assigned to *I.yokdon*.

## Materials and methods

Specimens were collected by beating shrub canopies and are deposited in Tay Bac University (TBU), Son La City, Vietnam.

All specimens are preserved in 75% ethanol. Female epigyna were cleared in a 10% potassium hydroxide (KOH) solution at room temperature for approximately 12 hours, then stored in small glass vials with the corresponding specimens. The left male palp was used for morphological descriptions and illustrations. Specimens were examined and illustrated using Olympus BX51 and SZ61 stereomicroscopes, with measurements obtained using an Olympus STM4 measuring microscope. Photographs were taken with a Sony A7 IV digital camera mounted on the Olympus BX51 and SZ61 microscopes. Image stacks were processed and edited using Adobe Photoshop 2024.

All measurements are given in millimetres (mm). Leg measurements are presented as total length, followed by segment lengths in parentheses (femur, patella + tibia, metatarsus, tarsus). Terminology used in the text and figure legends follows Patoleta et al. (2020).

The following abbreviations are used in the text and figures: **ALE** anterior lateral eye; **AME** anterior median eye; **BB** bulbus; **CD** copulatory duct; **CO** copulatory opening; **E** embolus; **ECP** epigynal coupling pocket; **FD** fertilisation duct; **GD** glandular duct; **PLE** posterior lateral eye; **PME** posterior median eye; **RTA** retrolateral tibial apophysis; **S** spermatheca; **SD** sperm duct.

## Taxon treatments

### Indopadilla yokdon

Hoang, Phan & Vo, 2024

25973E6A-7D50-5D9D-AB25-50FF69CE102B

Indopadilla yokdon Hoang et al., 2024

#### Materials

- **Type status:** Other material. **Occurrence:** catalogNumber: TBU-ARA-SAL-002.1 to 002.2; individualCount: 2; sex: 1 male, 1 female; lifeStage: adult; occurrenceID: 0939735E-A8FC-508A-BCED-215AAC1FE247; **Taxon:** scientificName: *Indopadillayokdon*; class: Arachnida; order: Araneae; family: Salticidae; genus: Indopadilla; specificEpithet: yokdon; scientificNameAuthorship: Hoang, Phan & Vo, 2024; taxonomicStatus: accepted; **Location:** country: Vietnam; countryCode: VN; stateProvince: Son La; locality: Ban Mong village; verbatimElevation: 860 m; verbatimLatitude: 21°18.223’N; verbatimLongitude: 103°51.304’E; verbatimCoordinateSystem: WGS84; **Event:** eventDate: May 29, 2023; eventRemarks: collected by Vu D.T.; **Record Level:** language: en; collectionCode: Arachnida; basisOfRecord: PreservedSpecimen
- **Type status:** Other material. **Occurrence:** catalogNumber: VNMN-SAL-078; individualCount: 1; sex: female; lifeStage: adult; occurrenceID: CA65BF4D-C1C2-5F92-A572-A9438CA7FA7B; **Taxon:** scientificName: *Indopadillayokdon*; class: Arachnida; order: Araneae; family: Salticidae; genus: Indopadilla; specificEpithet: yokdon; scientificNameAuthorship: Hoang, Phan & Vo, 2024; taxonomicStatus: accepted; **Location:** country: Vietnam; countryCode: VN; stateProvince: Dak Nong; locality: entrance of Co cave, Dray Sap special used forest; verbatimElevation: 379 m; verbatimLatitude: 12°31.819’N; verbatimLongitude: 107°53.387’’E; verbatimCoordinateSystem: WGS84; **Event:** eventDate: May 21, 2024; eventRemarks: collected by Pham D.S.; **Record Level:** language: en; collectionCode: Arachnida; basisOfRecord: PreservedSpecimen

#### Description

**Figure 1.**
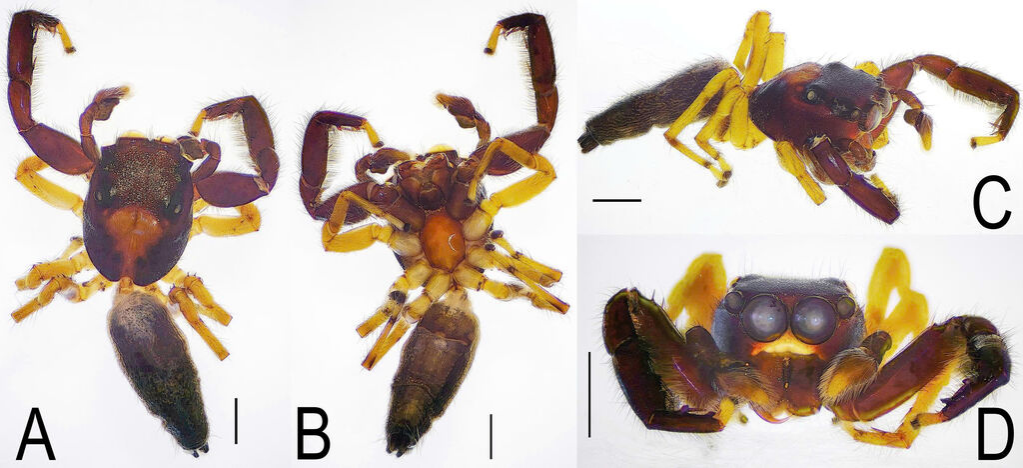
Male *Indopadillayokdon* (TBU-ARA-SAL-002.1), habitus. **A** Dorsal view; **B** Ventral view; **C** Right side view; **D** Frontal view of prosoma. Scale bars: 1.0 mm.

**Figure 2.**
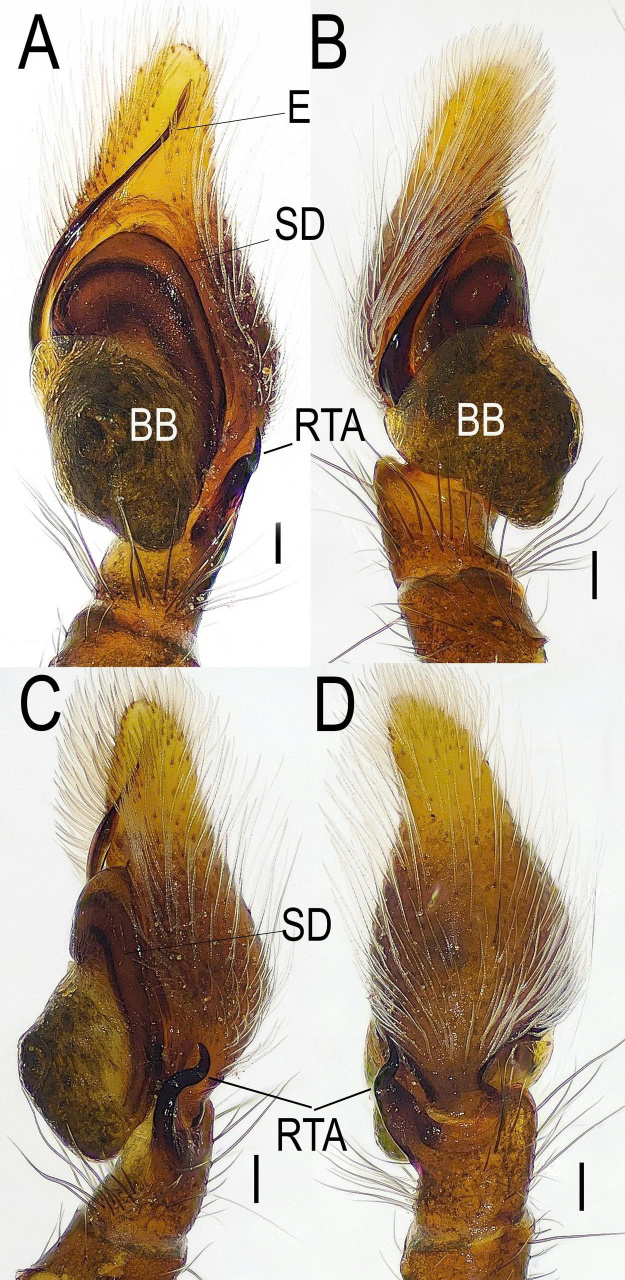
Male *Indopadillayokdon* (TBU-ARA-SAL-002.1), left palp. **A** Prolateral view; **B** Retrolateral view; **C** Left side view; **D** Dorsal view. Scale bars: 0.1 mm.

***Male*** (TBU-ARA-SAL-002.1) (Fig. 1A-D and Fig. 2A-D).

Total length 7.09. Carapace 3.0 long, 2.28 wide. Abdomen 3.75 long, 1.48 wide. Pedicel 0.34 long. Clypeus wide 0.4. Width of eye rows: anterior eye row 1.78; anterior medial eye row 1.3; posterior lateral eye row 1.33. Eye sizes and interdistances: AME 0.62, ALE 0.21, PME 0.08, PLE 0.24, ALE-PME 0.35, ALE-PLE 0.8. Legs' measurements: I 6.19 (2.1, 0.94, 1.56, 1.04, 0.55), II 4.5 (1.43, 0.76, 0.93, 0.9, 0.48), III 4.4 (1.43, 0.68, 0.73, 0.94, 0.62), IV 5.46 (1.35, 0.77, 1.26, 1.33, 0.75).

Carapace red-brown to dark brown, with a pale pan-shaped pattern extending from behind the posterior lateral eyes (PLE) to the posterior margin, clothed with sparse white setae. Area surrounding the posterior median eyes (PME) and PLE is black. Chelicera, endite, labium, sternum red-brown. Leg I (excluding its pale yellow tarsus) is reddish-brown and appears to be the largest and darkest amongst the four legs, while the remaining three are slender and uniformly pale yellow (Fig. 1). Femora of all legs bear a single prolateral macroseta centrally placed near the patella. Pedipalp similar in colour to leg I; cymbium yellow-brown to pale yellow. Embolus curved, unbranched, with a broad and plate-shaped base and a relatively slender distal part, extending from the mid-point of the bulbus to nearly the apex of the cymbium. Retrolateral tibial apophysis (RTA) distinctive, approximately three-quarters the length of the tibia in lateral view, pointed and curved in an S-shape (Fig. 2). Abdomen narrow and elongate, the width smaller than that of the carapace. Dorsum dark grey with four brown spots; venter light grey with two longitudinal dotted lines extending from the book lungs to the tracheal spiracle.

**Figure 3.**
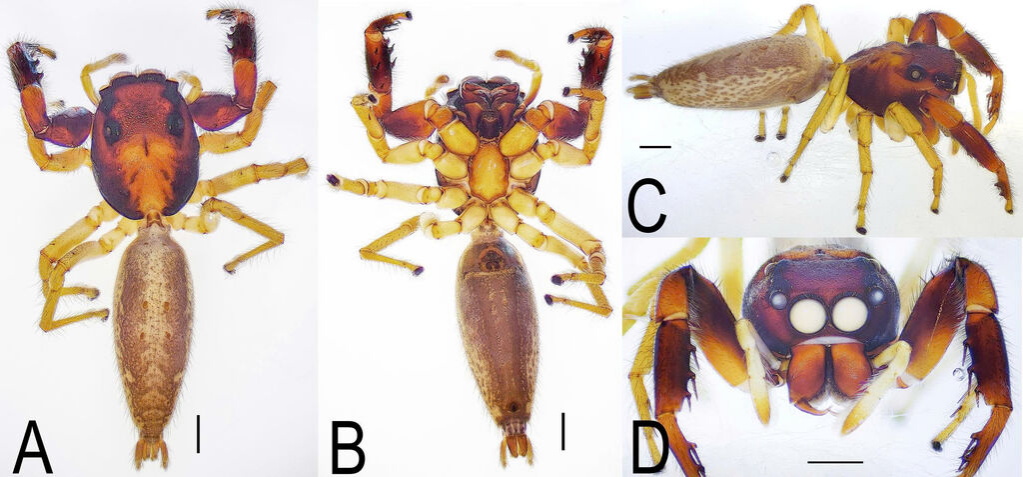
Female *Indopadillayokdon* (TBU-ARA-SAL-002.2), habitus. **A** Dorsal view; **B** Ventral view; **C** Right side view; **D** Frontal view of prosoma. Scale bars: 1.0 mm.

**Figure 4.**
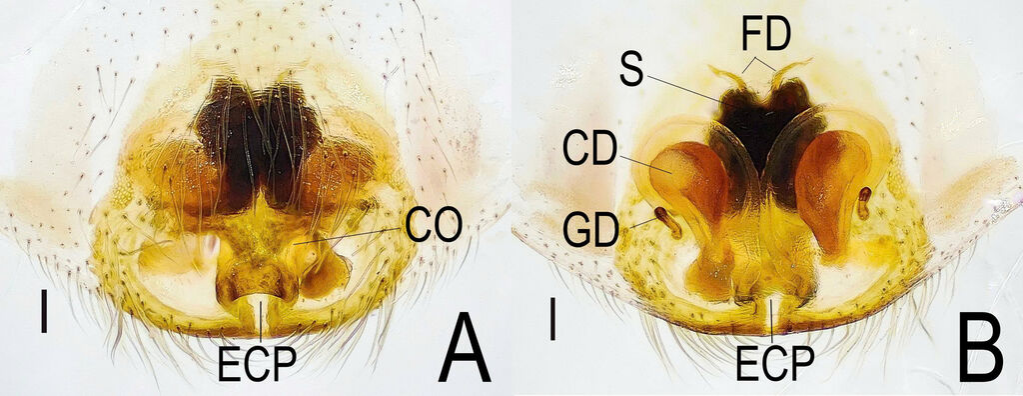
Female *Indopadillayokdon* (TBU-ARA-SAL-002.2), genital morphology. **A** Epigyne, ventral view; **B** Vulva, dorsal view. Scale bars: 0.1 mm.

***Female*** (TBU-ARA-SAL-002.2) (Fig. 3A-D and Fig. 4A-B).

Total length 10.62. Carapace 3.99 long, 2.98 wide. Abdomen 6.05 long, 2.15 wide. Clypeus height 0.43. Pedicel 0.58 long. Width of eye rows: anterior eye row 2.05; anterior medial eye row 1.43; posterior lateral eye row 2.41. Eye sizes and interdistances: AME 0.56, ALE 0.34, PME 0.08, PLE 0.31, ALE-PME 0.45, ALE-PLE 0.97. Legs measurements: I 7.93 (2.45, 1.48, 1.9, 1.4, 0.7), II 5.85 (1.97, 1.07, 1.14, 1.02, 0.65), III 5.79 (1.68, 1.11, 0.93, 1.22, 0.85), IV 7.36 (2.17, 0.91, 1.53, 1.93, 0.82).

Posterior eye row bordered in black. Clypeus pale. First pair of leg reddish-brown; remaining legs pale yellow. Pedipalps pale yellow. Abdomen oblong, narrower than carapace, dorsum pale grey with distinct herring-bone pattern and four small brown spots (Fig. 3A). Epigyne with large, bean-shaped copulatory ducts (Fig. 4B). The epigyne is distinctly longer than wide. The epigynal coupling pocket is deep, delicate and dorsally curved, forming a dome-shaped tunnel. Copulatory ducts are prominent, bean-shaped and accompanied posteriorly by small, short and clearly distinguishable glandular ducts. The spermathecae are complex, coiled and heavily sclerotised. Detailed descriptions of these structures are provided in Hoang et al. (2024).

#### Diagnosis

The male of *Indopadillayokdon* can be distinguished from all other congeners by the following combination of characteristics: (1) The embolus is plate-shaped, thin, curved, unbranched and extends from the mid-point of the bulbus to near the apex of the cymbium; (2) The retrolateral tibial apophysis (RTA) is distinctive, measuring approximately three-quarters the length of the tibia in lateral view; it is pointed and curved in an S-shape.

Characters shared with the female: The carapace is reddish-brown with a yellowish-brown funnel-shaped pattern on the posterior half (Fig. 1A and Fig. 3A). The clypeus white (Fig. 1D and Fig. 3D). The chelicera bear four teeth on the promarginal row. The first pair of legs is reddish-brown, while the remaining legs are light yellow. The femora of all legs bear lateral macroseta and an additional macroseta on the dorsal surface near the patella.

#### Ecology

Both specimens from Son La Province and the single specimen from Dak Nong Province were found inhabiting shrub canopies within tropical forest areas.

## Supplementary Material

XML Treatment for Indopadilla yokdon
